# Generation of musculoskeletal cells from human urine epithelium-derived presomitic mesoderm cells

**DOI:** 10.1186/s13578-024-01274-w

**Published:** 2024-07-15

**Authors:** Huiru Gao, Xingnan Huang, Zepo Cai, Baomei Cai, Kaipeng Wang, Junyang Li, Junqi Kuang, Bo Wang, Ziwei Zhai, Jin Ming, Shangtao Cao, Yue Qin, Duanqing Pei

**Affiliations:** 1 CAS Key Laboratory of Regenerative Biology, Guangzhou Institutes of Biomedicine and Health, Chinese Academy of Sciences, Guangzhou, 510530 China; 2 Laboratory of Cell Fate Control, School of Life Sciences, Westlake University, Hangzhou, 310024 China; 3 Guangdong Provincial Key Laboratory of Stem Cell and Regenerative Medicine, Guangzhou Institutes of Biomedicine and Health, Chinese Academy of Sciences, Guangzhou, 510530 China; 4 University of the Chinese Academy of Sciences, Beijing, 100049 China; 5 Guangzhou Laboratory, Guangzhou, 510000 China

**Keywords:** UiPSM cell, Differentiation, Skeletal myocytes, MYOD, Transplantation

## Abstract

**Background:**

Numerous studies have shown that somite development is a necessary stage of myogenesis chondrogenesis and osteogenesis. Our previous study has established a stable presomitic mesoderm progenitor cell line (UiPSM) in vitro. Naturally, we wanted to explore whether UiPSM cell can develop bone and myogenic differentiation.

**Results:**

Selective culture conditions yielded PAX3 and PAX7 positive skeletal muscle precursors from UiPSM cells. The skeletal muscle precursors undergo in vitro maturation resulting in myotube formation. MYOD effectively promoted the maturity of the skeletal myocytes in a short time. We found that UiPSM and MYOD mediated UiPSM cell-derived skeletal myocytes were viable after transplantation into the tibialis anterior muscle of MITRG mice, as assessed by bioluminescence imaging and scRNA-seq. Lack of teratoma formation and evidence of long-term myocytes engraftment suggests considerable potential for future therapeutic applications. Moreover, UiPSM cells can differentiate into osteoblast and chondroblast cells in vitro.

**Conclusions:**

UiPSM differentiation has potential as a developmental model for musculoskeletal development research and treatment of musculoskeletal disorders.

**Supplementary Information:**

The online version contains supplementary material available at 10.1186/s13578-024-01274-w.

## Introduction

**Fig. 1:**
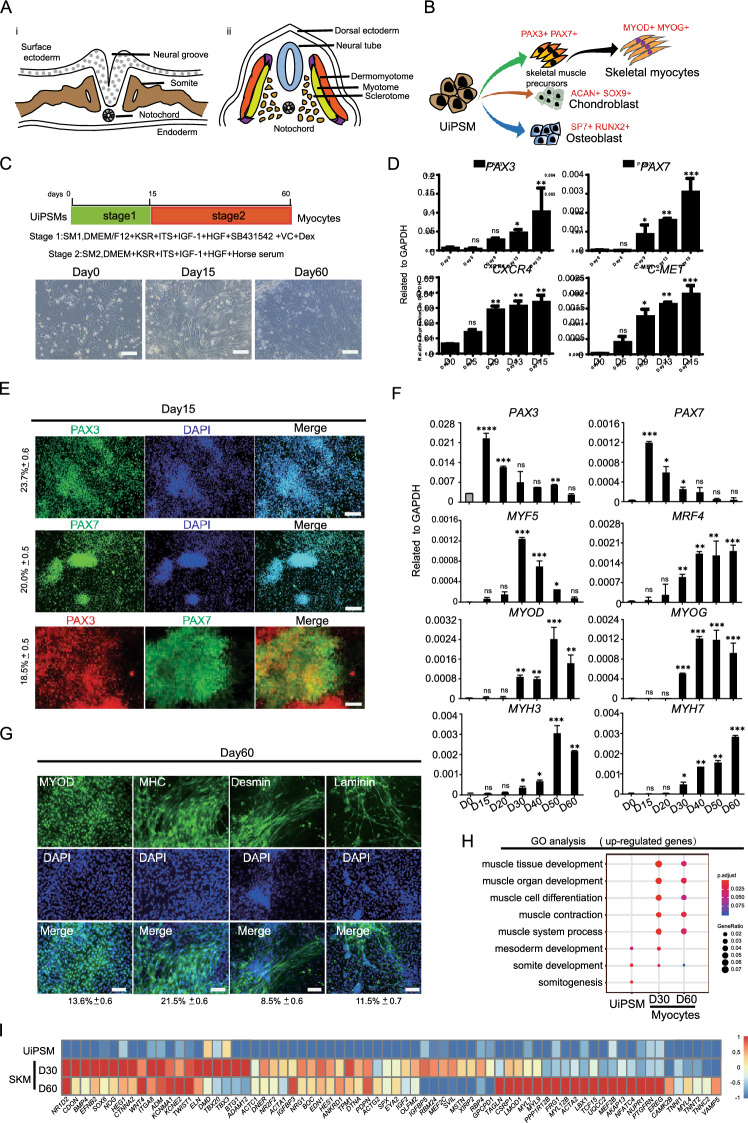
Differentiation of UiPSM cells into skeletal myocytes in vitro. **A** Schematic diagram of somite development. a. Illustration of the epithelial somite's spatial relationship to surrounding structure. b. Depiction of the differentiated somite's spatial relationship to surrounding structures. Dorsally, the somite differentiates into the dermomyotome and sclerotome. The dermomyotome subsequently gives rise to the myotome, which develops into skeletal muscle tissue. The sclerotome differentiates into osteoblasts and chondroblasts, forming the axial skeleton. **B** Schematic diagram of UiPSM cell differentiation into skeletal myocytes, osteoblasts and chondroblasts. **C** Schematic overview of stepwise differentiation of skeletal myocytes from UiPSM cells. Representative images show the morphological changes from UiPSM cells to skeletal muscle filaments. Scale bars, 100 µm. **D** Representative gene expression of human skeletal muscle satellite cells (*PAX3, PAX7, CXCR4, C-MET*) at day 15. Data are mean ± SD, n = 3 independent experiments. (*P ≤ 0.05). **E** Immunofluorescence of PAX3 and PAX7 during the differentiation of skeletal muscle cell form UiPSM at day 60 (left). The scale bar represents 100 µm. The values on the left represent the percentage of positive cells statistically. Data are mean ± SD, n = 3 independent experiments, each experiment counted 100 fields of view. **F** Representative gene expression of human skeletal muscle satellite cells (*PAX3, PAX7*) and skeletal myoblasts (*MYOD, MYOG, MRF4*) and skeletal myocytes (*MYH3, MYH7*) during the differentiation process. Data are mean ± SD, n = 3 independent experiments. (*P ≤ 0.05). **G** Immunofluorescence of MYOD, MHC, Desmin, Laminin during the differentiation of skeletal muscle cell form UiPSM at day 60 (left). The scale bar represents 100 µm. The following values represent the percentage of positive cells statistically. Data are mean ± SD, n = 3 independent experiments, each experiment counted 100 fields of view. **H** The UiPSM cells differentiated at day 30 and day 60 were enriched for GO terms of skeletal muscle development. **I** Heatmap illustrating the gene expression of skeletal muscle development related genes with dramatical change in UiPSM cell derived myocytes at day 30 and day 60

During the mammalian embryonic development, the paraxial mesoderm, situated bilaterally adjacent to the neural tube, forms during gastrulation. The anterior segment of this mesoderm epithelializes into segmented somites [1]. These somites subsequently compartmentalize along the dorsal–ventral axis into two distinct structures: a dorsal epithelial dermomyotome and a ventral mesenchymal sclerotome. The dermomyotome differentiates into skeletal muscle, brown adipose tissue, and the dermis of the back, whereas the sclerotome gives rise to the axial skeleton and tendons [2] (Fig. 1A).

Skeletal muscle, the largest tissue by mass in the body, is crucial for movement and support. However, millions of individuals worldwide are suffering from skeletal muscle atrophy, caused a range of factors, including cachexia, sarcopenia, and muscular dystrophies. The latter factors, encompassing over 30 distinct genetic disorders, often result in paralysis and are frequently associated with cardiopulmonary complications [3]. Currently, treatments are predominantly conservative and lack definitive cures. The replacement of diseased muscle with healthy muscle fibers derived from stem cells offers a promising approach. Prior research has shown that myogenic progenitors, which arise during myogenesis, can be differentiated directly or reprogrammed from embryonic stem cells or pluripotent stem cells (PSCs) [4–8]. However, the incomplete differentiation of PSCs poses a clinical safety risk. The process of in vitro PSC myogenesis necessitates a transition through a mesodermal stage [9]. For therapeutic applications, the generation of abundant, engraftable, tissue-specific cells are required. Establishing a mesodermal progenitor cell lineage with specific differentiation capabilities to expand the pool of transplantable myocytes is an area warranting further investigation.

In our research, we have successfully reprogrammed human urinary epithelial cells into presomitic mesoderm progenitor cells (UiPSMs). These cells are capable of long-term expansion in vitro and differentiate into mesoderm cell types in vivo, displaying anteroposterior axis and segmentation clock features [10]. In this study, we established a UiPSM-based approach to skeletal myogenesis, enhanced by MYOD-mediated maturation. Transplantation of these cells into injured mouse muscle demonstrated sustained regeneration and repair. Additionally, we induced differentiation into osteoblasts and chondrocytes (Fig. 1B). These findings open new pathways for treating musculoskeletal diseases.

### Generation of human skeletal muscle satellite cell from UiPSM

Myogenesis during development contains two distinct phases: initially, the Paired Box Homeotic Gene (Pax7) and its homolog Pax3 confer myogenic fate in an early embryonic or primary phase [11]; subsequently, the cell fusion and the incorporation of myonuclear from proliferating Pax7+ progenitors facilitate secondary myogenesis [12]. Consequently, we devised a two-stage protocol for differentiating UiPSM cells into skeletal myocytes (Fig. 1C). Previous study had proved that inhibiting endogenous TGF-β (using LDN93189 or SB431542) and stimulating myogenesis-promoting factors such as hepatocyte growth factor (HGF) and insulin-like growth factor 1 (IGF1) are crucial for maximal Pax7 induction [13]. And dexamethasone (DEX) has been shown to improve myogenesis, advances muscle structure, and increases force production in engineering skeletal muscle tissue [14]. Building on this foundation, through high-throughput screening and testing of various small molecule combinations, we found that Vitamin C effectively enhance myogenic differentiation. Thus, for the initial phase, the optimal combination for maximal PAX7 induction from UiPSM cells in 15 days involved activating IGF/HGF alongside Vitamin C and Dexamethasone, and inhibiting TGF-β with SB431542. We termed this medium SM1. The cytokine receptor CXCR4, in tandem with the adaptor protein Gab1, which mediates c-Met signaling, regulates the development of migrating Pax3-positive myogenic progenitor cell [15, 16]. Due to their functional roles, *Cxcr4* and *c-Met* enable the isolation of high-purity PAX3+ PAX7+ skeletal muscle precursors [17]. RT-PCR analysis confirmed that after 15 days of differentiation, UiPSM cells robustly expressed *PAX3, PAX7, C-MET*, and *CXCR4*, verifying the formation of PAX3 and PAX7 positive skeletal muscle precursors (Fig. 1D). Immunofluorescence assays revealed 30–50% of cells were PAX3+ or PAX7+, with 30% being PAX3+ PAX7+, indicating muscle lineage commitment (Fig. 1E).

For secondary myogenesis, Pax7+ and Pax3+ myogenic precursors derived from UiPSM cells fused to form larger fibers, expressing more mature MyHC isoforms, like fast MyHC [18]. We transitioned UiPSM cells, after 15 days in SM1 medium, to a medium supplemented with HGF, IGF-1, and horse serum for up to 2 months to promote further differentiation (Fig. 1C). During dermomyotome formation, cells begin downregulating Pax3 and Pax7, concurrently activating muscle regulatory factors (MRFs) including Myf5, MyoD (Myod1), and MRF4 (Myf6) [9, 19]. Our qPCR results showed a decrease in *PAX3* and *PAX7* expression by day 15, alongside increased expression of *MYF5, MYOD*, and *MRF4* by day 30, suggested the activation of a myogenic transcription factor network (Fig. 1F). Notably, Myf5, transiently expressed in dermomyotome cells, is known to specify the skeletal muscle lineage in mouse and chicken embryos [9, 20]. Its expression peaked on day 30, indicating the onset of myogenesis (Fig. 1F). Myogenin, functioning downstream of Myf5 and Myod, controls the terminal differentiation of myoblasts into myocyte [20, 21]. The concurrent activation of MYOG at day 30 gave supported to the formation of myocytes (Fig. 1F). Afterwards, Myogenin-positive myocytes formed embryonic myosin heavy chain-positive skeletal myocytes, eventually fusing into primary myofibers [22, 23]. The increase in *MYH3* and *MYH7* transcripts between days 40 and 60 signified terminally differentiated skeletal myocytes/myotubes (Fig. 1F). Terminal differentiation was further evidenced by the expression of additional mature muscle markers, Desmin, Myosin Heavy Chain (MHC), and Laminin, in approximately 10–20% of cells by day 60, as shown by immunocytochemistry (Fig. 1G). RNA-seq data analysis indicated that UiPSM-differentiated cells on days 30 and 60 were closely related to muscle tissue development and muscle contraction, enriching for skeletal muscle (*ACTC1, ACTA1, ACTA2*), Myosin Light Chain (*MYL7, MYL9, MYL4*, and *MYL12B)*, and Dystrophin (*DMD*) genes (Fig. 1H, I), supported a mature myocyte phenotype. Therefore, at this stage, UiPSM-derived PAX3+ and PAX7+ myogenic precursors had differentiated into proliferating MYOD1+ myoblasts, MYOG+ myocytes, and MHC+ myocytes/myotubes.

### MYOD increased the maturity of skeletal myocytes

Given the modest efficiency of myogenic conversion in UiPSM cells, these cells might not be effectively activating Muscle Regulatory Factors (MRFs), which are crucial for initiating downstream muscle-specific gene expression. MyoD, a pioneering factor in myogenesis, is known for its ability to convert various cell types, including fibroblasts, pigment cells, nerve cells, adipocytes, and hepatocytes, into skeletal muscle [24, 25]. Prior studies have shown that MyoD-reprogrammed hESCs can efficiently generate myotubes by activating myogenic programs [6, 26]. Consequently, we hypothesized that MYOD might be capable of reprogramming the nuclei of UiPSM cells into a skeletal muscle phenotype.

**Fig. 2:**
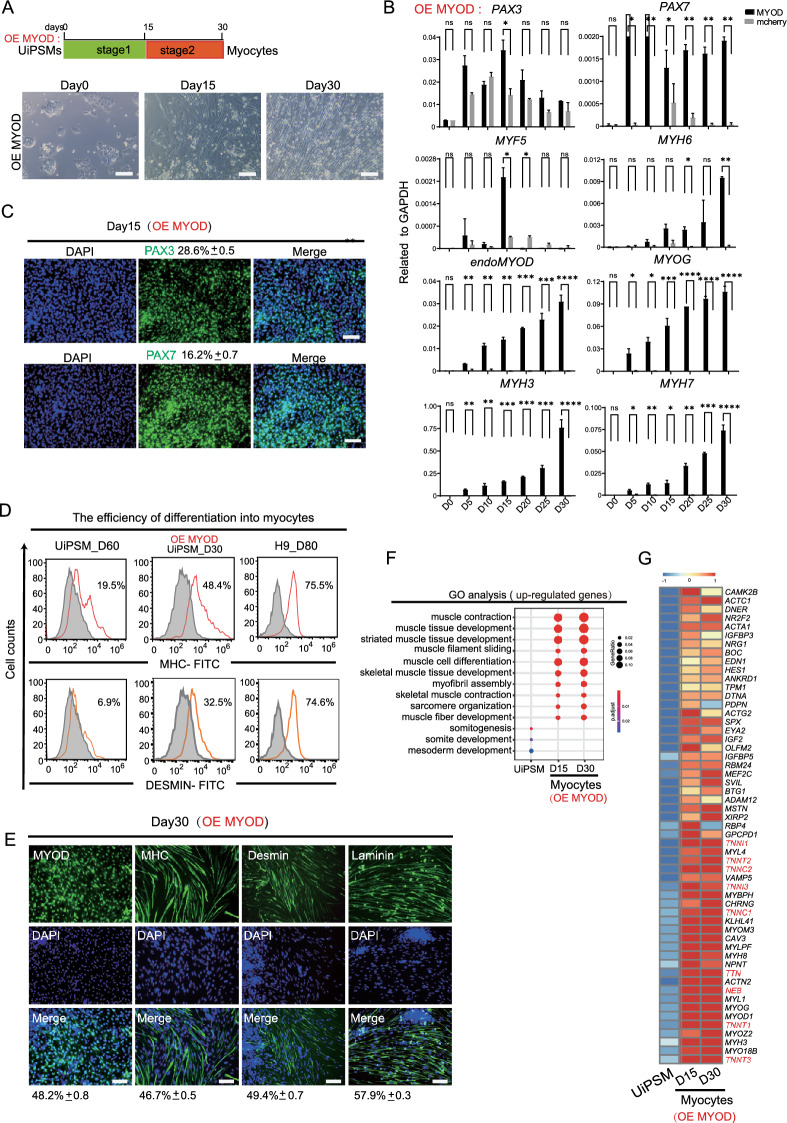
MYOD promoted the maturity of skeletal myocytes in vitro. **A** Schematic overview of stepwise differentiation of skeletal myocytes from UiPSM with ectopic MYOD. SKM: skeletal muscle cells. Representative images show the morphological changes from UiPSM cells to skeletal myocytes. Scale bars, 100 µm. n = 3 independent experiments. **B** Representative gene expression of human skeletal muscle satellite cells (PAX3, *PAX7*), skeletal myoblasts (*MYOD, MYOG, MRF4*) and skeletal myocytes (*MYH3, MYH7*) when overexpressed ectopic MYOD during the differentiation process. Mcherry as a negative control of overexpression vector. Data are mean ± SD, n = 3 independent experiments. (*P ≤ 0.05). **C** Detection of skeletal muscle satellite cell-specific genes (*PAX3* and *PAX7*) in MYOD-mediated differentiated UiPSM cells at day 15. The following values indicate the percentage of positive cells statistically (Data are mean ± SD, n = 3 independent experiments). Scale bars, 100 µm. **D** Flow cytometric analysis evaluating differentiation efficiency via MHC and Desmin protein expression in skeletal muscle cells at day 60 of differentiation. hESC (H9)-derived skeletal muscle cells at day 85 are used as a positive control. **E** Immunofluorescence analysis of MYOD, MHC, Desmin, and Laminin in UiPSM-derived muscle fibers at day 30 with ectopic MYOD. Scale bar represents 100 µm. The values indicate the percentage of positive cells statistically (Data are mean ± SD, n = 3 independent experiments, each experiment counted 100 fields of view). Scale bars, 100 µm. **F** MYOD-mediated differentiation of UiPSM cells into skeletal muscle cells at days 15 and 30, showing enrichment for skeletal muscle development-related GO terms. **G** Heatmap illustrating gene expression changes specific to skeletal myocytes in MYOD-mediated UiPSM cell-derived myocytes at days 15 and 30

To test this hypothesis, we evaluated the ability of UiPSM cells to undergo direct myogenic conversion in response to ectopic MYOD expression under consistent culture conditions (Fig. 2A). Remarkably, MYOD expression significantly increased the expression of myocyte-specific genes, such as *MYOG, MYH3,* and *MYH7*. The expression of the myoblast gene *MRF4* and endogenous *MYOD* increased approximately tenfold. In contrast, MYOD expression did not notably activate early myogenesis genes *PAX3, PAX7*, and *MYF5* (Fig. 2B, Additional file 1: Fig. S1A). This outcome is consistent with the understanding that *MyoD* acts downstream of *Pax3, Pax7,* and *Myf5* in the myogenic cascade [22, 27, 28], and *MYOD* was also shown to be insufficient to induce Pax3-positive cells [23]. Additionally, *Myf5* and *MyoD* are differentially distributed in the epaxial and hypaxial dermomyotome, respectively [27]. These findings suggested that MYOD can effectively induce later stages of myogenesis in UiPSM cells. The efficacy of MYOD in driving terminal myogenesis in UiPSM cells was further substantiated by both FACS and immunocytochemistry. These methods revealed a quick increase in the expression of mature muscle marker proteins—Desmin, Myosin Heavy Chain (MHC), and Laminin.

Notably, the expression of these markers escalated to approximately 50% by day 30 (Fig. 2D, E). RNA sequencing (RNA-seq) data analysis provided deeper insights into the nature of MYOD-induced myogenesis on days 15 and 30. The data indicated a progression towards more mature muscle features, encompassing aspects such as muscle contraction, sarcomere organization, striated muscle tissue development, muscle filament sliding, myofibril assembly, and muscle fiber development (Fig. 2F). A notable enrichment of skeletal muscle genes, including *ACTC1, ACTA1, ACTG2,* and *ACTN2*, was observed. Additionally, the expression of Myosin Light Chain (MYL) genes (*MYL4, MYL1*, and *MYLP*) and components of the troponin complex (*TNNI1, TNNT2, TNNC2, TNNI3, TNNC1, TNNT1, TNNT3*) associated with the sarcomere thin filament in striated muscle was significantly enhanced [29, 30] (Fig. 2G). These findings collectively suggested that MYOD efficiently accelerates the maturation of skeletal myocytes derived from UiPSM cells within a short time (Fig. 2D).

To further assess the sructural and functional mature of the UiPSM- and iMYOD UiPSM-derived skeletal myocytes, we collected samples of UiPSM direct differentiation and exogenous MYOD-mediated differentiation at day 30 to detect myofiber diameter and the resting membrane potential (RMP). Myotube diameter is a conventional measurement of muscle mass [31, 32]. MyoD-mediated skeletal muscle differentiation resulted in an increase in diameter, and the myotube number also showed a significant increase (Fig. S2A-C). These increases suggest enhanced myogenic differentiation.The resting membrane potential (RMP) of a cell is crucial for transitioning from a resting state to an excitable state, allowing the cell to perform its proper function. Hence, we evaluated the RMP of differentiated cells at day 30. The RMP recorded from UiPSM directly differentiated cells at day 30 was relatively low, with a mean value of − 15.77 ± 4.16 mV. In contrast, UiPSM cells overexpressing MYOD (myocytes) displayed a higher RMP, with a mean value of − 60.27 ± 2.92 mV (Fig. S2D, E).Typically, the resting membrane potential of human myoblast cell lines (AB1167) is − 74.1 ± 0.8 mV [33]. Similar RMP mean values are found in human ES-derived (− 72.25 ± 4.57 mV) and iPS-derived (− 72.12 ± 2.55 mV) myocytes [34]. We detected no significant difference between MYOD-differentiated cells and the reported myocytes using a t-test (Fig. S2E). These results collectively support that MYOD promotes UiPSM cell differentiation to a mature myotube state by day 30.

Recent advancements have demonstrated MYOD's efficiency in converting hESCs and other cell types into skeletal myocytes in vitro [5, 6, 23, 35]. However, when comparing MYOD-reprogrammed hESCs (H9) with UiPSM cells, the former showed lower myogenic efficiency on day 15 (Additional file 1: Fig. S3A, B). Notably, human urine cells (UCs) failed to activate myogenesis (Additional file 1: Fig. S3D). These results suggested an inherent predisposition of UiPSM cells towards skeletal muscle differentiation, surpassing that of hESCs. PAX7, recognized for its role in generating myogenic progenitors from hPSCs [4, 36], was less effective in our studies. Overexpression of PAX7 did not significantly induce *MYOD* and *MRF4* expression in either UiPSM or hESC (H9) cells on day 15 under identical conditions (Additional file 1: Fig. S3C, E), indicating that PAX7 alone might not be sufficient to trigger myogenesis in these cell types. In conclusion, these data suggested that MYOD is uniquely capable of efficiently directing UiPSM cells into more mature skeletal myocytes within a relatively brief period.

### UiPSM induced skeletal myocytes contribute to muscle regeneration

**Fig. 3:**
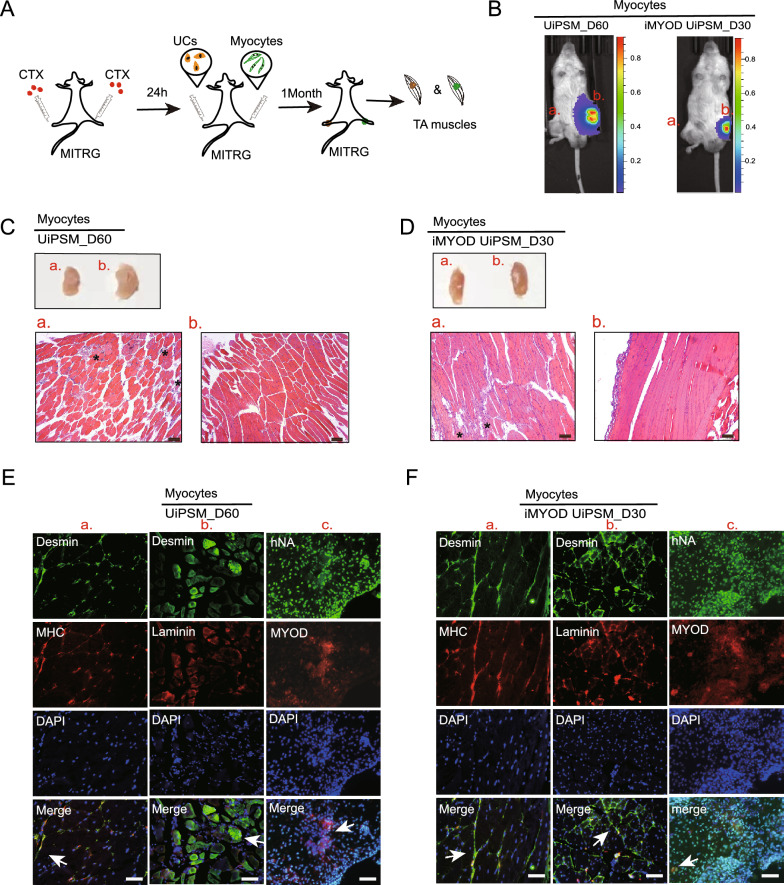
Transplantation of UiPSM and iMYOD UiPSM cells derived human myocytes in muscle injury model. **A** Schematic overview of the transplantation methodology for UiPSM and iMYOD UiPSM cell-derived human skeletal myocytes into the TA muscle of MITRG mice, following treatment with cardiotoxin (CTX) for 24 h. Urine cells serve as a negative control and are transplanted into the left tibialis anterior muscle. **B** Bioluminescence imaging (BLI) signal captured at the right tibialis anterior graft site in a representative MITRG mouse treated with CTX, 1 month after transplantation. UCs transplanted into the left TA muscle in (a), as a negative control. UiPSM derived myocytes transplanted into the rright TA muscle in (b). **C** Morphological characteristics of TA muscle tissue in after transplanted UiPSM-derive myocytes and UCs. H&E staining of longitudinal sections of TA muscles showed the aggregation of inflammatory factors (asterisk) could still be seen locally in the left tibial anterior muscle after transplanting UCs in (a). H&E staining of longitudinal sections of TA muscles after transplanted UiPSM differentiated into myocytes at day 60 in (b). Scale bars, 100 µm. **D** Morphological characteristics of TA muscle tissue in after transplanted iMYOD UiPSM-derive myocytes and UCs. H&E staining of longitudinal sections of TA muscles showed the local aggregation of inflammatory factors (asterisk)in the left tibial anterior muscle after transplanting UCs in (a). H&E staining of longitudinal sections of TA muscles after transplanted iMYOD UiPSM-derived myocytes at day 30 in (b). Scale bars, 100 µm. **E** TA muscle from UiPSM-derived myocytes evaluated for the expression of myocyte-specific markers. Longitudinal section showed the colocalization of Desmin (DES) and Myosin Heavy Chain (MHC) (arrows in (a.)). Transversal section showed the colocalization of Desmin and Laminin (arrows in (b.)). Transversal section showed the colocalization of human nuclei antibody (hNA) and MYOD (arrows in (c.)). Scale bars, 100 µm. **F** TA muscle from iMYOD UiPSM-derived myocytes evaluated for the expression of myocyte-specific markers. Colocalization of Desmin (DES) and Myosin Heavy Chain (MHC) (arrows in (a.), and colocalization of Desmin and Laminin (arrows in (b.)) were shown in Longitudinal sections. Transversal section showed the colocalization of human nuclei antibody (hNA) and MYOD (arrows in (c.)). Scale bars, 100 µm

The potential of MyoD-reprogrammed hPSCs and hPSC-derived myoblasts for engraftment upon transplantation into mouse muscle has been established in previous studies [8, 37–40]. To further explore the myogenic potential of UiPSM cells in vivo, an appropriate animal model is necessary. In our study, we transplanted UiPSM and iMYOD UiPSM derived skeletal myocytes into the tibial anterior (TA) muscle of the MITRG mouse, which is an immune-deficient strain like NSG commonly used as a recipient of human hematopoietic cells [41]. Before transplantation, both UiPSM-derived skeletal myocytes and hUCs were transduced with a lentiviral vector carrying luciferase and green fluorescent protein (eGFP) [42]. This approach allows for noninvasive monitoring of cell survival in vivo through bioluminescence imaging (BLI). To induce muscle necrosis, we injected 8 µg of cardiotoxin (CTX) bilaterally into the TA muscle of thirteen MITRG mice. Twenty-four hours after CTX injection, we transplanted UIPSM-derived, MYOD-mediated differentiation to day 30 or no MYOD-mediated differentiation to day 60 skeletal muscle cells into damaged TA muscle. This was done to assess the survival and regenerative capability of these human cells in the mouse model (Fig. 3A). For blank control group, one mouse received CTX in the right hindlimb TA muscle and saline solution in the left hindlimb. The remaining twelve mice were split into two groups. One group received injections of luc+ (luciferase-expressing) UiPSM directly differentiated skeletal myocytes into the right hindlimb TA muscles. Another group received luc+ UiPSM-derived skeletal myocytes with MYOD expression in the right hindlimb TA muscles. As a control, the contralateral TA muscles, which were also pre-injured with CTX, were injected with human urine cells (UCs).

One month after cardiotoxin injury and subsequent grafting, the mice exhibited a stable bioluminescence imaging (BLI) signal in the right hindlimb. This signal was indicative of graft survival of both UiPSM and iMYOD UiPSM cell-derived skeletal myocytes. In contrast, the left tibialis anterior muscle of the same mice, injected with luciferase-expressing urine cells (luc + UC), showed no BLI signal, thereby underscoring the robust long-term survival of the transplanted skeletal myocytes in the host muscle (Fig. 3B). The aggregation of inflammatory factors could still be seen locally in the left tibial anterior muscle by H&E staining (Fig. 3C, D), also suggested UiPSM and iMOD UiPSM cells derived skeletal myocytes repaired the damaged muscle. Further validation of engraftment and contribution to muscle regeneration was achieved through immunohistochemical analysis. Muscle sections were stained with antibodies specific to Desmin, Myosin Heavy Chain (MHC), Laminin, MYOD, and Human Nuclear Antigen (hNA) (Fig. 3E, F). The expression of these markers in the muscle tissue provided strong evidence that the UiPSM-derived skeletal myocytes were contributing to its regeneration. An important observation was the absence of tumor formation in the transplanted mice, even over a longer period of 2 months.

### UiPSM derived skeletal myocytes mature after transplantation

**Fig. 4:**
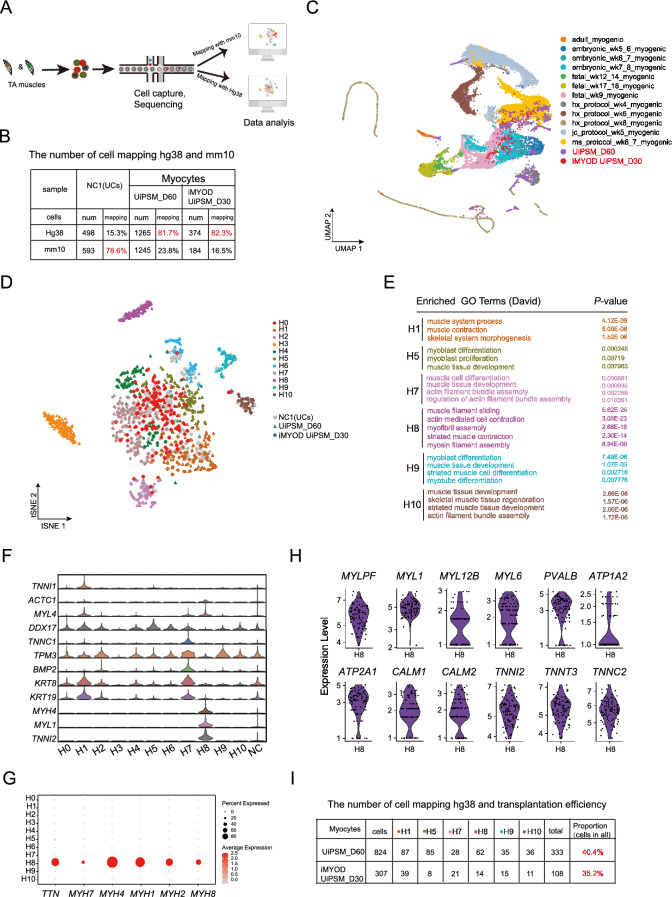
scRNA-seq analysis of the cell components of the human skeletal myocytes derived grafts. **A** Schematic overview of the process involving transplantation of UiPSM and iMYOD UiPSM-derived human myocytes and urine cells into injured mouse TA muscles. One month after transplantation, TA muscles were dissociated into single cells for RNA sequencing. Sequencing data were then mapped to the human (hg38) and mouse (mm10) genomes, respectively. **B** Quantification of cell numbers and mapping rates from the sequencing data. **C** UMAP representation of scRNA data from Xi et al.(2020) and our studies, including eight sources of myogenic. **D** t-SNE plot showed the projection of 2137 cells mapping to human genome (hg38). These cells could be divided in 11 color-coded clusters. The circles, triangles and diamonds represent the control group (urine cells), and UiPSM and iMYOD UiPSM derived human myocytes, respectively. **E** Gene ontology (GO) analysis revealed gene expression profiles of the groups (H1, h5, h7, h8, h9, h10) that are highly associated with skeletal muscle development in the above 11 clusters. p value < 0.05. **F** Violin plot showing the expression and ratio of typical mature skeletal muscle cells related genes in each cluster. **G** Dot plot of expression levels for titin (TTN) and different Myh genes. Color indicates expression level in each cluster; Dot size percent of cells expressing selected genes in each cluster. **H** Violin plot of expression levels for calcium-handling gene PVALB, Calcium-Transporting ATPase Sarcoplasmic Reticulum (ATP1A2, ATP2A1), Calmodulin (CALM1, CALM2) and troponin (TTN, TNNI2, TNNT3, TNNC2). **I** Calculating the percentage of cells in skeletal myocytes related clusters (H1, h5, h7, h8, h9, h10) on all sequenced cells of the grafts from UiPSM and iMYOD UiPSM derived human myocytes, when mapping with human genome (hg38)

To comprehensively analyze the cell diversity at the transplanted sites (Fig. 3A), single-cell RNA sequencing (scRNA-seq) was performed on UiPSM and iMYOD UiPSM cells-derived skeletal myocytes, as well as the control urine cells, engrafted into the TA muscles after 1 month. Considering the presence of both human and mouse cells in the grafts, the scRNA-seq data were aligned to both human and mouse genomes accordingly (Fig. 4A). Of the 4159 individual cells analyzed, the experimental group showed more than 80% genome matching with the human genome and less than 20% with the mouse genome. In contrast, the control group exhibited nearly 80% genome matching with the mouse genome and only 15% with the human genome (Fig. 4B). This data indicated that both UiPSM and iMYOD UiPSM-derived skeletal myocytes not only survived but also actively participated in muscle regeneration post-transplantation into the damaged TA muscle.

To gain a comprehensive view of UiPSM-derived myocytes close to human skeletal muscle cell ontogeny, we evaluated the scRNA-seq data of UiPSM derived myocytes after transplantation and reported human myocytes, including SKM cluster in scRNA-seq data of human limb muscle tissues from embryonic (week 5–8), fetal (week 9–18) as well as postnatal juvenile (year 7–11) and adult (year 34–42) stages [43], most of the cells in our lab overlapped with embryonic (week 7–8), fetal (week 9) myocytes (Fig. 4C). Typically, the primary generation fibers with central nuclei are present in the human skeletal muscle at week 8 of gestation [44]. Human fetal skeletal muscle (week 9) enriched for muscle contraction, mitochondria and oxidative phosphorylation (OxPhos) as well as calcium signaling [43]. These suggested UiPSM derived mature skm state after transplantation. At the same time, we also compared the data of UiPSM derived myocytes after transplantation with SkM cells generated from hPSCs in representative protocols [22, 35, 43, 45], we only found a large group of cells coincide with MS protocol during week 6–7 differentiation, and a small group of cells match to SKM cells in HX protocol at week 4 (Fig. 4C). A small subset of terminal differentiating myoblasts-myocytes (MBMC) were observed in MS protocol at week 6–7, and half of SKM cells were PAX7-GFP^+^ populations in HX protocol at week 4 [43]. These suggested UiPSM derived terminal differentiating after transplantation, and retained a group of PAX7 positive populations. Furthermore, a small group of cells neared to adult (year 34–42) myogenic cells (Fig. 4C), confirmed a mature SKM state in UiPSM derived myocytes after transplantation.

Upon detailed examination, the scRNA-seq data from human skeletal myocyte grafts could be categorized into 11 distinct clusters when mapped to the human genome (hg38). Moreover, the data UiPSM and iMYOD UiPSM cell derived skeletal myocytes showed similar distribution (Fig. 4C, D), suggested skeletal myocytes in different levels of maturity showed an almost uniform ability to differentiate in the body in vivo. Cluster H1 enriched for muscle contraction and muscle system process related genes, *TNNI1, MYL4, DDX17, TPM3, KRT8* and *KRT18.* Cluster H5 was specifically associated with myoblast differentiation and proliferation, highly enriched for *DDX17, TPM3*, *KRT8* and *KRT18.* Actin filament bundle assembly related genes, *TNNC1, TPM3*, *BMP2, KRT8* and *KRT18,* were enriched in Cluster H7. Cells in cluster H8 highly expressed striated muscle contraction related genes, *MYL4, MYH4, MYL1* and *TNNI2*. And cells in cluster H9, H10 enriched for muscle tissue development and myotube differentiation and highly expressed *TPM3* (Fig. 4E, F). These supported variety of skeletal muscle cell types existed in UiPSM derived myocytes after transplantation.

Skeletal muscles, known as striated muscles, in vertebrates are primarily composed of populations of fast-type and slow-type muscle fibers [46]. Notably, cells in cluster H8 represented a major mature group of skeletal myocytes, highly expressing fast Myh (fMyh) genes, including Myh2, Myh1, and Myh4, while expressing low levels of slow Myh7 and neonatal Myh8 (Fig. 4G) [46], This suggested that a mature population of myonuclei is closely related to fetal muscle. Most myosin light chains are also expressed in developing skeletal muscle [46]The enrichment of MYLPF, MYL1, MYL12B, and MYL6 further supports that cells in H8 are similar to fetal muscle. Typically, striated muscles are regulated by Ca2+, which is released from the sarcoplasmic reticulum (SR) and binds to troponin (Tn) on the actin filament [47]. Most Myh4 myonuclei express the calcium-handling gene PVALB [48]. High expression of PVALB, Calcium-Transporting ATPase Sarcoplasmic Reticulum (ATP1A2, ATP2A1), Calmodulin (CALM1, CALM2) and troponin (TTN, TNNI2, TNNT3, TNNC2) (Fig. 4G, H), supported the functional diversity of striated muscle. This finding substantiates that UiPSM and iMYOD UiPSM cell-derived skeletal myocytes adopt a more mature myocyte fate in vivo. Additionally, the analysis revealed that almost 40% of the UiPSM and iMYOD UiPSM cell-derived skeletal myocytes survived, with almost 12–14% (in H8) contributing to maturation post-transplantation (Fig. 4G).

To further understand the composition of the transplanted grafts, particularly the 20% of cells that matched the mouse genome (mm10), we analyzed the cell populations derived from the tibialis anterior muscle of the transplanted mice. Through single-cell RNA sequencing, this subset of cells was classified into 11 distinct clusters. The cells in clusters m0, m3, m5, m6, m8, and m9 were predominantly associated with various muscle-related functions and processes, as indicated by Gene Ontology (GO) enrichment analysis. These functions included skeletal muscle contraction, muscle cell differentiation, striated muscle hypertrophy, actin filament bundle assembly, and musculoskeletal movements. These clusters showed high expression of mouse skeletal muscle markers such as *Actg1, Tnni2, Tnnt3, Tnnt2, Tnnt1* (Additional file 1: Fig. S4A-D). This expression profile suggested that the 20% of cells indeed originated from the mice's tibialis anterior muscle. Collectively, these findings confirm that UiPSM cells -derived skeletal myocytes are capable of surviving in vivo and actively contributing to muscle regeneration.

### Generation of the human skeletal cell from UiPSM cells

**Fig. 5:**
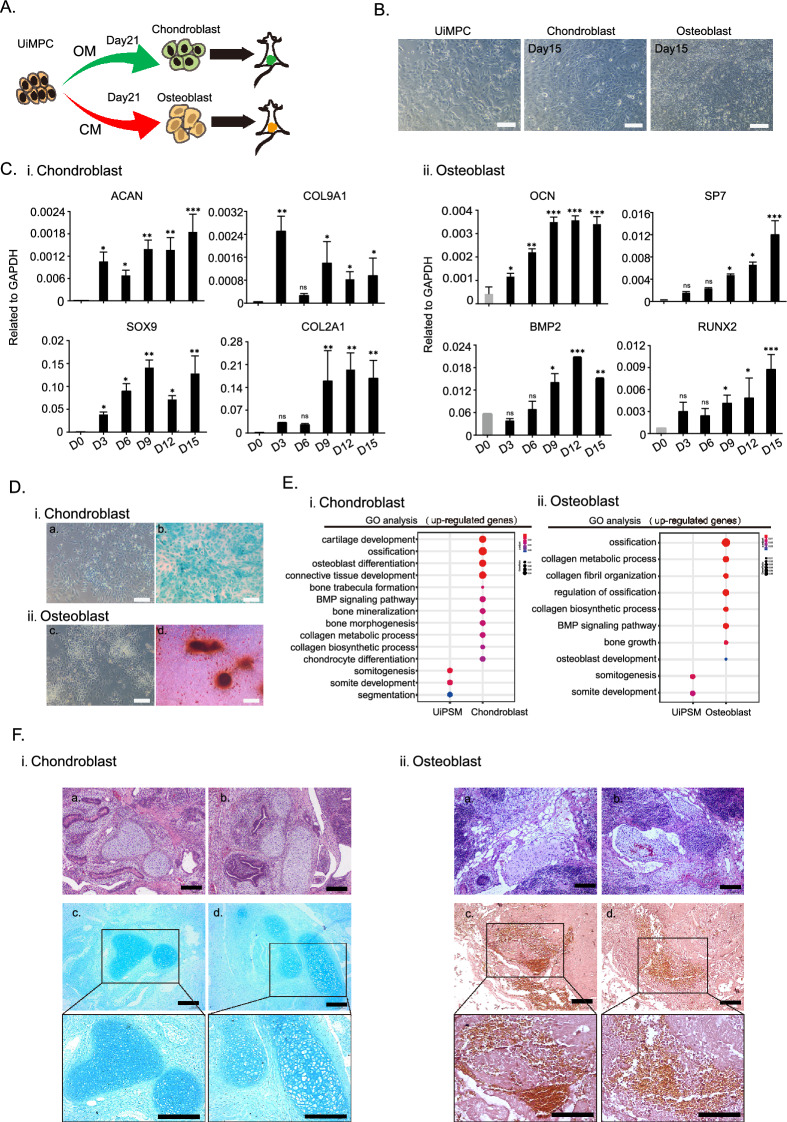
UiPSM cells derived axial osteogenesis. **A** Schematic overview of chondroblasts and osteoblasts differentiated from UiPSM. **B** Representative images show the chondroblasts and osteoblasts differentiated from UiPSM cells at day 15. Scale bars, 100 µm. n = 3 independent experiments. **C** i. Representative gene expression of chondroblast (*ACAN, COL9A1, SOX9, COL2A1*) during the differentiation process. ii. Representative gene expression of osteoblast (*OCN, SP7, BMP2, RUNX2*) during the differentiation process. (Data are mean ± SD, n = 3 independent experiments). **D** i. Representative images show the Alcian blue staining of UiPSM cells and chondroblasts at day 15. a, UiPSM cells as a negative control, could not be dyed; b. UiPSM cells induced Chondroblast could be dyed blue in Alcian blue staining. Scale bars, 100 µm. ii. Representative images show the alizarin red staining of UiPSM cells and osteoblasts at day 15. c, UiPSM cells as a negative control, could not be dyed; d. UiPSM cells induced Osteoblast could be dyed red in alizarin red staining. Scale bars, 100 µm. **E** i. Representative gene ontology enrichment terms associated with chondroblast differentiation in UiPSM and chondroblast cells at day 15. ii. Representative gene ontology enrichment terms related to osteoblast differentiation in UiPSM and osteoblast cells based on up-regulated genes in each sample. **F** a, b H&E staining revealing the morphological features of grafts derived from UiPSM chondroblasts and osteoblasts transplanted into MITRG mice after 1 month. c, d These sections were further characterized by Alcian Blue and Alizarin Red staining, respectively. Scale bars, 100 µm

Vertebrate skeletons comprise craniofacial, limb, and axial bones, each emanating from distinct embryonic lineages, with axial bones deriving from paraxial mesoderm cells (somites) [49]. This study aimed to further explore the potential of UiPSM cells to differentiate into cartilage and bone (Fig. 5A). Mainly referring to the established protocols for hPSC differentiation into osteoblasts and chondroblasts [50, 51], we formulated TGF-β3-dependent chondrogenic induction medium and BMP2-dependent osteoblast induction medium, termed ‘CM’ and ‘OM’ media, respectively (Fig. 5A, B). On day 15, chondroblast-specific genes (*ACAN, COL2A1, SOX9, COL9A1*) and osteoblast-related genes (*BMP2, RUNX2, BGLAP, SP7*) were notably upregulated in ‘CM’ and ‘OM’ media, respectively (Fig. 5C), indicating differentiation of UiPSM cells toward cartilage and bone fates. Alcian Blue and Alizarin Red staining of chondroblasts and osteoblasts, respectively, confirmed tissue-specific characteristics (Fig. 5D). RNA-seq data analysis verified the identities of osteoblast and chondroblast cells (Fig. 5E, Additional file 1: Fig. S5A). To further evaluate the differentiation ability of UiPSM cells-derived osteoblasts and chondroblasts in vivo, we subsequently introduced UiPSM cells -derived osteoblasts and chondrocytes into immunodeficient MITRG mice. After 1 month, the harvested paraffin sections of the grafts, subjected to the H&E staining, demonstrated a prevalence of chondrocytes and osteoblasts (Fig. 5F, Additional file 1: Fig. S5B), indicating the differentiation potential of UiPSM cells into cartilage and bone in vivo.

## Discussion

Consistent with the in vivo development of presomitic mesoderm [9, 11, 52], UiPSM cells are capable of differentiating into skeletal myocytes, osteoblasts, and chondroblasts in vitro. Our prior work has shown that UiPSM cells, reprogrammed from urinary cells (UCs) and exiting the pluripotent state, retain somatic stemness, thereby mitigating the risk of tumorigenesis [10]. This suggested the potential of UiPSM cells to serve as a developmental model for musculoskeletal research. UiPSM-based systems will also provide exciting opportunities to further our understanding of the regulatory mechanisms governing skeletal muscle genesis during human development.

Furthermore, both UiPSM and iMYOD-reprogrammed UiPSM-derived skeletal myocytes, exhibiting varying maturity levels, displayed a uniformly robust capacity of differentiation in vivo. We postulate that post-transplantation; the internal environment must maintain a certain equilibrium. It is also conceivable that the methodology used to process the TA muscle for single-cell isolation was excessively robust, potentially leading to a relatively homogenous cell population. Further evidence is needed to support these observations. Morever, the intervention of single-cell RNA-seq approach helping to reveal the ability of UiPSM and iMYOD UiPSM cells-derived skeletal myocytes to repair damaged muscle tissue. This approach promises to contribute to a comprehensive understanding and the establishment of novel therapeutic strategies for skeletal muscle diseases, such as muscular dystrophies and age-related regenerative deficits.

Moreover, UiPSM cells provide an experimental model system bearing resemblance to embryonic stages and offer distinct advantages: the ease of sourcing initial cells coupled with their unique characteristics enables the generation of patient- or disease-specific PSM cells, essential for their effective application. The establishment of a self-renewing UiPSM cell line with the potential for presomitic differentiation permits an in-depth examination of human embryogenesis events (myogenesis and axial osteogenesis). Ultimately, the field of UiPSM cell-derived skeletal myogenesis and axial osteogenesis is inceptive, presenting opportunities and possibilities for developmental biology, drug discovery, and regenerative medicine.

## Material and methods

### MITRG mice

MITRG [41] mice are genetically modified to be Rag2-deficient and Il2rg-deficient and express human cytokines (M-CSFh, IL-3/GM-CSFh, TPOh) crucial for developing innate immune cells. This makes them highly receptive to human hematopoiesis, facilitating the complete development and function of human myeloid cells such as monocytes, macrophages, and NK cells derived from liver or adult CD34+ progenitor cells. This humanized mouse model is widely used in research involving human cell engraftment and patient-derived tumor xenografts (PDX).

### Cell lines

#### UiPSM cell line

Refer to the paper [10]. Human urine derived epithelioid cells were electrical transduced unintegrated plasmids (pEP4EO2SET2K and pCEP4-miR-302–367 cluster) using Amaxa™ Basic Nucleofector™ Kit (Lonza, VPI-1005), then cultured 7–12 days in the induction medium (IM: advanced DMEM/F12 (Gibco, 12,634–010) supplemented with 3 µM CHIR99021 (Synthesized in GIBH), 10 ng/ml bFGF (PeproTech P09038), 5 ng/ml EGF (R&D systems, 236-EG) and 5 µM EPZ5676 (Selleck Chemicals S7062)), changed the medium every 2 days. The UiPSM colonies can be digested with 0.25% Trypsin–EDTA (Gibco, 25200056), and passaged at intervals of 5 days, seeded at a density of 1 × 10^5^ cells per well in 12-well plate coated with Matrigel, maintained with defined medium (DM: advanced DMEM/F12 supplemented with 3 µM CHIR99021, 10 ng/ml bFGF, 5 ng/ml EGF and 1 µM A8301 (R&D systems,2939).

### Human ES (H9)

Human ES (H9) cells obtained from ATCC cell banks, were cultured in mTeSR (STEM CELL, 85850) on Matrigel (Corning, 354277)-coated cell culture plastics and passaged using accutase (STEM CELL, 07922).

#### Generation of skeletal muscle cells from UiPSMs

UiPSMs were dissociated into single cells using 0.25% Trypsin–EDTA (Gibco, 25200056) and then seeded sparsely (7.5 × 10^4 cells) onto new Matrigel-coated 24-well plates, maintained overnight in UiPSM medium. Myogenic differentiation was induced in two stages:

Stage I: The SM1 medium, used for 15 days, comprised DMEM/F12 enriched with 15% KSR (Gibco, 10828028), 1% ITS (Gibco, 41400045), 1% NEAA, 0.1 µM ß-ME, 4 ng/ml IGF-1 (Pepro Tech, 250-19), 10 ng/ml HGF (R & D systems,294-HG-250), 3 µM CHIR99021, 50 ng/ml VC (Sigma-Aldrich, 49752), 0.5 ng/ml Dexamethasone Phosphate disodium (Dex, Target Mol, T0947L), and 2 nM SB431542. Media was changed every 2 days.

Stage II: The SM2 medium, used until the presence of skeletal muscle fiber bundles, included DMEM supplemented with 15% KSR, 2% Horse serum (Gibco, 16050122), 1% NEAA, 0.1 µM ß-ME, 4 ng/ml IGF-1, and 10 ng/ml HGF. Media was changed every 3 days.

#### Generation of skeletal muscle cells from hESCs

As described in published paper [23]. H9 were digested into single cells using Accutase and sparsely passaged 3 × 10^4^ onto new Matrigel-coated cell 24-well plate 2 days for subsequent induction. for subsequent myogenic differentiation, was initially induced via a modified approach including five stages including DiCL for 3 days, DiCLF for 3 days, DKHIFL for 2 days, DKI for 4 days, and DKHI for last induction as Jérome Chal described. DiCL: DMEM/F12 supplemented with 15% KSR, 1% ITS, 1% NEAA, 3 µM CHIR99021 and 500 nM LDN193189; DiCLF: DMEM/F12 supplemented with 15% KSR, 1% ITS, 1% NEAA, 3 µM CHIR99021, 500 nM LDN193189 and 20 ng/ml bFGF; DKHIFL: DMEM/F12 supplemented with 15% KSR, 1% NEAA, 0.1 µM ß-ME, 2 ng/ml IGF-1, and 10 ng/ml HGF, 500 nM LDN193189 and 20 ng/ml bFGF; DKI: DMEM/F12 supplemented with 15% KSR, 1% NEAA, 0.1 µM ß-ME, 2 ng/ml IGF-1; DKHI: DMEM/F12 supplemented with 15% KSR, 1% NEAA, 0.1 µM ß-ME, 2 ng/ml IGF-1, and 10 ng/ml HGF.

#### Generation of osteoblast from UiPSMs

UiPSMs were passaged at a density of 7.5 × 10^4 onto new Matrigel-coated 24-well plates and incubated overnight for sclerotome induction. The basal medium used was DMEM, supplemented with 10% FBS (NTC, SFBE), 50 ng/ml vitamin C, 100 nM β-Glycerophosphate (PeproTech, 154804–51-0), and 1 µM 1-Thioglycerol (Sigma, 96-27-5). The medium was refreshed every 3 days over a period of 15 days.

#### Generation of chondroblast from UiPSMs

For chondroblast induction, UiPSMs were similarly passaged onto Matrigel-coated 24-well plates. The culture medium comprised DMEM with 10% FBS, 1% ITS, 1% S/P, 50 ng/ml vitamin C, 0.1 nM β-Glycerophosphate, 4 ng/mL TGF-β3 (PeproTech, 100-36E), and 20 ng/ml BMP2 (PeproTech, 500-P195). This medium was also replaced every 3 days for a duration of 15 days.

#### Immunofluorescence staining

Cells were fixed with 4% formaldehyde for 30 min (TM) and washed thrice with PBS. They were then permeabilized and blocked in perm/blocking buffer (PBS + 0.1% Triton X-100 (Sigma, 9002-93-1) and 3% BSA (Sigma, 9048–46-8)) for 1 h (TM). Overnight staining at 4 °C followed using primary antibodies diluted in blocking buffer (PBS with 3% BSA). After three PBS washes, cells were stained with the appropriate secondary antibody for 1 h (diluted in perm/blocking buffer). For nuclear counterstaining, DAPI was used (1:5000 dilution) for 3 min before final washes and fluorescent microscopy. We used the antibodies including the antibody of PAX3, PAX7, MYOD, Myosin Heavy Chain (MF20), Laminin a3 and Desmin.

#### Quantitative RT-PCR

Total RNAs were isolated from cells with TRIzol and converted into cDNAs with HiScript II Q RT SuperMix (Vazyme, R222-01) for qPCR, and then q-PCR analyzed with specific q-PCR primers and stained with ChamQ SYBR qPCR Master Mix ((Vazyme, R311-02)).

#### Intracellular flow cytometry

Adherent cell populations were briefly washed with DPBS to remove dead or floating cells, dissociated with 0.25% Trypsin–EDTA, suspend the digestion with 10%FBS, centrifugated to prepare cell suspensions, and washed with DPBS twice. Subsequently, cells were fixed in 100 µl 4%PFA (20 min, TM), washed twice in DPBS; The cold final concentration of 100% methanol to 90% methanol was slowly added to the precooled cells through mild vortex mixing to permeate the cells at least 10 min on the ice, washed twice in DPBS. The cells were resuspended in a 100 µl diluted primary antibody (1:100) with blocking buffer, incubate at room temperature for 1 h, washed twice in DPBS; The cells were resuspended in a 100µL diluted secondary antibody (1:500) with blocking buffer and incubate at room temperature for 30 min, washed twice in DPBS; Cells were resuscitated in 200–500 l DPBS and analyzed on Accuri C6 Plus. Data analysis was done using FlowJo7.6.1. software (LLC). The antibodies involved Mouse monoclonal anti-Myosin Heavy Chain (MF20) (R & D systems, MAB4470) and Rabbit polyclonal anti-Human Desmin (R & D systems, abs106139).

#### Measurement of myotube diameter

Images of the myotubes were acquired at 100 × magnification before the contractile force assay. The myotube diameters were measured in all cells from 10 random fields of view per substrate using NIS-Elements (Nikon). The width of the widest position of the myotube and moved back and forth by 50um were measured in a cell, the mean of the three values was the diameter of the myotube.

#### Electrophysiology

Methods mainly referred to Xinle Zou’s protocol [53].Myocytes were dissociated into single cells using the STEMdiff Cardiomyocyte Dissociation Kit (Stemcell Technologies, #05025). Action potentials (APs) were triggered at 1 Hz by 3 ms suprathreshold stimulations. Briefly, APs were recorded at room temperature (RT) using the whole-cell patch clamp technique by an Axopatch 700A amplifier and Digidata 1550B digitizer (Axon Instruments, Foster City, CA, USA). External solution contained the following: 132 mM NaCl, 4.8 mM KCl, 2 mM CaCl2, 1.2 mM MgCl2, 10 mM HEPES, and 5 mM glucose (pH was adjusted to 7.4 with NaOH). Internal solution contained the following: 110 mM KCl, 5 mM ATP-K2, 11 mM EGTA, 10 mM HEPES, 1 mM CaCl2, and 1 mM MgCl2 (pH was adjusted to 7.3 with KOH). Pipette series resistance was typically 1.5–3 MΩ when filled with internal solution.

#### Transplantation of skeletal muscle cells

Preparing male MITRG mice of 4–6 weeks of age. The CTX (8 µg/g, WHIGA, 9012-91-3) was injected into the anterior tibialis, after 24 h treatment, urine cells were transplanted into the left side as negative control, and the skeletal muscle cells derived from UiPSM cells were transplanted to the right side to observe the activity in vivo. The UiPSM cells were labeled with lenti-luciference plasmid before the differentiation. The skeletal muscle cells derived from UiPSM at day 60 and day 30 with exogenous MYOD digested with the solution including equal volume of 0.25% Trypsin–EDTA and 0.01% collagenase IV (Gibco, 17104019) for preparing of single cell suspension. The urine cells derived from UiPSM digested with 0.25% Trypsin–EDTA for preparing of single cell suspension. After centrifugation and pelleting, 1.5 × 10^6^ cells were resuspended in a small volume (50–100 µL) of cold DMEM/F12, counted, and diluted with an equal volume of cold Matrigel, yielding a cell suspension in a 1:1 mixture of DMEM-F12/Matrigel that was kept on ice until transplantation. Finally, the preparing cells were intramuscular injected into the anterior tibia.

#### Immunofluorescence in frozen sections

Fresh tissues isolated from mice were fixed in paraformaldehyde more than 24 h, the tissues were dehydrated with 20% sucrose until the tissue blocks sink to the bottom, continued to dehydrate with 30% sucrose 72 h. Finally, the tissue blocks was embedded with OCT (Solarbio, 4583), frozen in liquid nitrogen and stored in the -80℃ refrigerator, which were used to make frozen slices. The frozen sections fixed with 4% formaldehyde (Electron Microscopy Services, in PBS) for 20 min (TM) and washed three times (PBS). Next, cleaning the surface of the slides and drawing a circle around the tissue with Pap pen. They were then permeabilized and blocked-in perm/blocking buffer (PBS + 0.1% Triton X-100 [Sigma] + 3%BSA for 1 h (TM) and then stained overnight (4 °C) with primary antibody diluted in blocking buffer (PBS + 3%BSA) in the wet box. Subsequently, the slides were washed three times (PBS) and stained with appropriate secondary antibody (diluted in perm/blocking buffer) for 1 h (TM). For nuclear counterstaining, the slides were stained with DAPI (1:5000, diluted in perm/blocking buffer) for 3 min and then washed twice more prior to conducting fluorescent microscopy. Rabbit polyclonal anti-Human MYOD (Cell signaling Technology, D8G3), Mouse monoclonal anti-Myosin Heavy Chain (MF20) (R & D systems, MAB4470), Mouse monoclonal anti-Human Laminin a3 (R & D systems, MAB2144), Rabbit polyclonal anti-Human Desmin (R & D systems, abs106139), Mouse monoclonal anti-Human HNA (Millipore, MAB1281).

#### Bioluminescence imaging

To non-invasively image luciferase + Human urine cell-derived donor cells after UiPSM clone, UiPSC clone and sclerotome lineage transplants, bioluminescence imaging was conducted. In brief, mice were injected intraperitoneally with 1.5 mg D-luciferin (ThermoFisher, L2916) in 100 µL volume of PBS 20 min prior to imaging. 5 min before imaging, mice were anesthetized by isoflurane and placed in the IVIS Spectrum imaging chamber. Data were subsequently analyzed using Living Image software.

#### Alcin blue dye staining for UiPSM-derived chondrocyte

The cartilage progenies stain with1 to access production of acidic proteoglycans as a key trademark of cartilage phenotypic function. UiPSM-derived chondroblast were stained with Alcin blue dye with standard procedures according Alcin blue dye staining kit (GENMED, GMS80015.1). Namely, UiPSM-derived chondroblast at day 15 of differentiation were fixed (with 4% formaldehyde, for 20 min at room temperature), washed twice in PBS, stained with Alcin blue dye solution for 1 h, washed with PBS, and wide-field visualization was performed with a Digital slice scanning and application system (Motic).

#### Alizarin red dye staining for UiPSM-derived chondroblast

The osteogenic induction process enables calcium ions to precipitate in the form of calcium salts, known as "calcium nodules". Alizarin red staining is often used to identify calcium nodules. UiPSM-derived osteoblast were stained with Alizarin red dye with standard procedures according Alizarin red dye staining kit (GENMED, GMS80017.1). Namely, UiPSM-derived chondroblast at day 15 of differentiation were fixed (with 4% formaldehyde, for 20 min at room temperature), washed twice in PBS, stained with Alizarin red dye solution for 15 min, washed with PBS, and wide-field visualization was performed with a Digital slice scanning and application system (Motic).

#### In vivo* transplantation to generate human ectopic bones*

UiPSM cells, tagged with lenti-luciferase and differentiated into day 5 sclerotome, were harvested using a brief PBS wash and dissociation with 0.25% Trypsin–EDTA. After centrifugation and pelleting, the cells were resuspended in a small volume of cold DMEM/F12, counted, and diluted with an equal volume of cold Matrigel to form a 1:1 DMEM-F12/Matrigel suspension. This mixture was kept on ice until used for subcutaneous transplantation into 1–2-month-old immunodeficient MITRG mice. Each graft consisted of approximately 1.5 × 10^6 sclerotome cells.

#### Histological analysis of ectopic bone grafts

The fresh tissue mass isolated from mice were fixed in paraformaldehyde more than 24 h, aftersubcutaneous transplantation of sclerotome progenitors ~ 1–2 months. Then embedded in paraffin, and sectioned. Slides with tissue sections were deparaffinized through sequential washes in histologicalgrade xylene (Sigma) and ethanol, and were stained with hematoxylin & eosin (as per standard procedures), Alcin blue dye (GENMED) and Alizarin red dye (GENMED).

### High throughput data collection and analysis

#### Bulk-population RNA-seq libraries construction

For bulk-population RNA-seq, RNA samples with a high RNA integrity (RIN) value were used for RNA-seq. Purified total RNA was capture with magnetic bead with oligo-polyA, interrupt and reverse-transcribed into cDNA using the VAHTSTM mRNA-seq V3 Library Prep Kit for Illumina (Vazyme, NR611,), purified using VAHTS DNA Clean Beads (Vazyme, N411). Next, cDNAs ligated adapters and amplified libraries using the amplification mix (Vazyme, NR611). Finally, sequencing the cDNAs library to obtain 150 bp paired-end reads.

#### Bulk RNA-seq and expression analysis

RNA-seq was preprocessed as described in published paper [54]. In brief, reads were aligned to a transcriptome index generated from the Ensembl annotations (v81, hg38), using RSEM27 and bowtie228 (—bowtie2—bowtie2-sensitivity-level very_sensitive—no-bam-output—estimate-rspd) and normalized using EDAseq29. Differentially expressed genes were filtered to select only those with log2 fold change greater than 2. RNA-seq data was expressed in units of GC-normalized tag counts.

#### Transcription factor motif discovery and gene ontology

Transcription factor motif analysis was performed by using HOMER230 with settings (-p 4 -size given). Gene ontology enrichment analysis was performed using clusterProfiler31.

#### Dissociating cells of grafts when transplanted UiPSM and iMYOD UiPSM cell-derived myocytes

UiPSM and iMYOD UiPSM cells -derived Skeletal myocytes were transplanted in TA muscles for a month. The TA muscles were immersed in DMEM with 0.1% penicillin–streptomycin on ice when isolated from the mice, digested with the same way like the teratoma tissue, while digestive solution including equal volume of 0.25% Trypsin–EDTA (Gibco) and 0.01% collagenase IV. Finally, cells were resuspended with DPBS containing 0.04% BSA (Sigma) to give a final concentration of 800 cells/ul.

#### Single-cell RNA-seq libraries construction

The cells underwent a brief wash with DPBS, were dissociated using Accutase (Stem Cell, 07992), and strained through a 100 µm filter (Solarbio, F8190) twice. After pelleting, they were resuspended in DPBS with 0.04% BSA to reach a final concentration of 800 cells/µl. For single-cell capture, this cell suspension was loaded onto a 10 × Chromium Chip A Single Cell Kit (10 × GENOMICS, 120236). The capture process was verified on a 10 × Chromium Controller, followed by cell lysis, reverse transcription, and cDNA synthesis. Post-amplification, the cDNA libraries were fragmented, amplified with adapters (Chromium Multiplex kit 10 × GENOMICS, PN-120262), and prepared for deep sequencing on Nova seq at Novogene company.

#### Analysis of single-cell RNA-seq

E mapped single-cell sequencing data to human genome by using CellRanger 3.0.2 with parametes ‘–localcores = 10–localmem = 3–mempercore = 3–maxjobs = 10–r1-length = 26–r2-length = 151’. We processed mapped data by using Seurat15 [19]. The count matrix was generated and excluded low quality cells (cells with less than 200 genes) and low-quality genes (genes expressing in less than 10 cells). The filtered count matrix was normalized by SCTransform in Seurat website, briefly SCTransform() regressed out mitochondrial mapping percentage, normalized, scaled and find highly variable genes (HVG). The top 5000 HVG were used for principal component analysis (PCA) and we picked first 50 principal components (PC) to perform downstream analysis. t-SNE (t-distributed Stochastic Neighbor Embedding) was performed using RunTSNE() in Seurat package.

### Data and code availability

The data supporting the conclusions of this Article. The UiPSM and UiPSC derived tissues scRNA-seq data are available at GEO under accession GSE185137.The bulk seq data of the UiPSM derived skeletal muscle cells with or without MYOD, osteoblast and chondrocyte are available at GEO under accession GSE185137. The scRNA-seq data of anterior tibial muscle when transplanted UiPSM derived skeletal muscle cells with or without MYOD are available at GEO under accession GSE185137.

### Quantification and statistical analysis

For counting the proportion of positive cells in immunofluorescence results. we randomly selected five horizons is each batch experiment, a total of 100 cells, and calculated the proportion of positive cell. We performed such experiments and statistics for a total of 3 times.

Data are presented as mean ± s.d. or mean ± s.e.m. as indicated in the figure legends. Unpaired twotailed Student’s t test, were used to assess statistically significant. P value < 0.05 was considered as statistically significant. No statistical method was used to predetermine the sample size. The experiments were not randomized. The investigators were not blinded to allocation during experiment and outcome assessment.

### Supplementary Information

**Additional file 1: Fig. S1**. The expression of ectopic MYOD in UiPSMs. **Fig. S2**. The ectopic MYOD expressed in hESC (h9) and hUCs and the ectopic PAX7 expressed in hESC (h9) and UiPSMs. **Fig. S3**. scRNA-seq analysis of the transplanted TA muscles when mapping mouse genome. **Fig. S4**. Transplantation of UiPSM cells-derived chondroblasts and osteoblasts in MITRG mice. **Table S1**. Listing the information of antibodies, chemicals and recombinant protein related to Experimental Procedures. **Table S2**. Primers used for qPCR.

## Data Availability

The data that supports the findings of this study are available in the method part and Additional file 1.

## Abbreviations

hPSCs: Human pluripotent stem cell
UiPSMs: Human urine cells induced presomitic mesoderm progenitor cells
IGF-1: Insulin-like growth factor 1
HGF: Hepatocyte growth factor
DEX: Dexamethasone
MRFs: Muscle regulatory factors
MHC: Myosin heavy chain
UCs: Human urine cells (epithelioid)
hESCs: Human embryonic stem cells
CTX: Cardiotoxin
iMYOD: Overexpress MYOD
eGFP: Green fluorescent protein
BLI: Bioluminescence imaging
TA muscle: Tibial anterior muscle
H&E staining: Hematoxylin & eosin staining
scRNA-seq: Single-cell RNA seq sequencing
hg38: Human genome (hg38)
mm10: Mouse genome (mm10)
GO: Gene ontology
