# Supplementary material for: Generation of musculoskeletal cells from human urine epithelium-derived presomitic mesoderm cells
Source: Cell Biosci. 2024 Jul 15;14:93. doi: 10.1186/s13578-024-01274-w (PMC11251367; doi:10.1186/s13578-024-01274-w)
Supplement: Supplementary file 1 — Additional file 1: Fig. S1. The expression of ectopic MYOD in UiPSMs. Fig. S2. The ectopic MYOD expressed in hESC (h9) and hUCs and the ectopic PAX7 expressed in hESC (h9) and UiPSMs. Fig. S3. scRNA-seq analysis of the transplanted TA muscles when mapping mouse genome. Fig. S4. Transplantation of UiPSM cells-derived chondroblasts and osteoblasts in MITRG mice. Table S1. Listing the information of antibodies, chemicals and recombinant protein related to Experimental Procedures. Table S2. Primers used for qPCR. [file 13578_2024_1274_MOESM1_ESM.pdf]

Figure S1

A

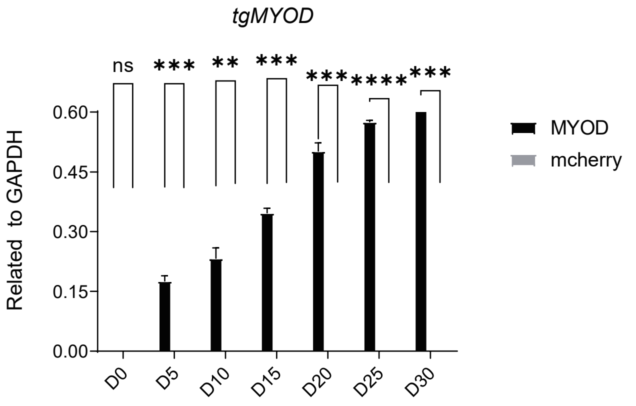
tgMYOD
ns
***
**
***
***
****
***
0.60
0.45
0.30
0.15
0.00
Related to GAPDH
D0
D5
D10
D15
D20
D25
D30
MYOD
mcherry

Figure S1 The expression of ectopic MYOD in UiPSMs

A. Representative gene expression of ectopic expressed *MYOD (tg MYOD)* during the iMYOD UiPSM cell-derived myogenesis process. Mcherry as a negative control of overexpression vector. Data are mean ± SD, n = 3 independent experiments.

Figure S2

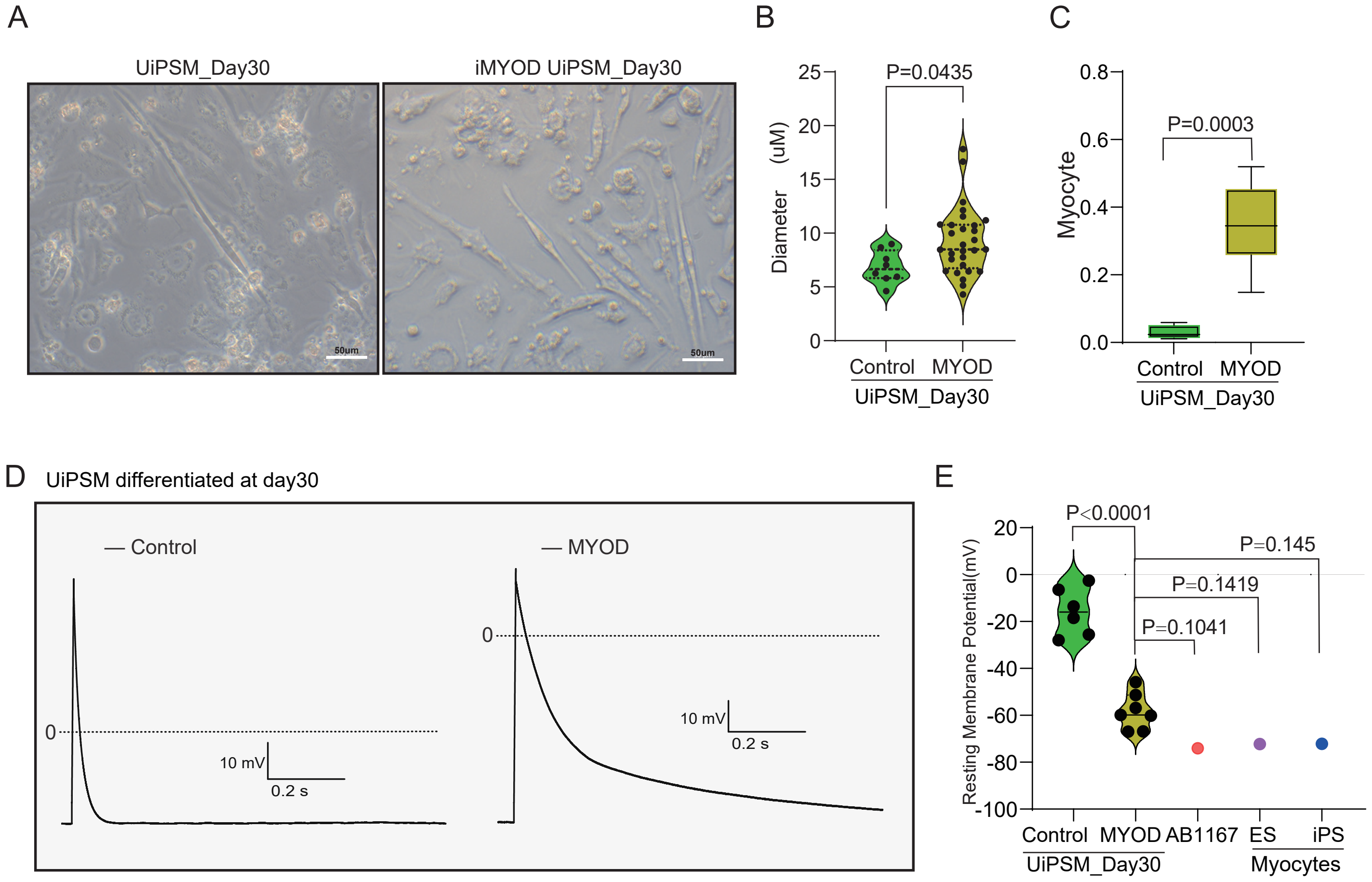

A
UiPSM_Day30
iMYOD UiPSM_Day30
50um
50um
B
P=0.0435
Diameter (uM)
25
20
15
10
5
0
Control
MYOD
UiPSM_Day30
C
P=0.0003
Myocyte
0.8
0.6
0.4
0.2
0.0
Control
MYOD
UiPSM_Day30
D
UiPSM differentiated at day30
— Control
— MYOD
0
10 mV
0.2 s
E
P<0.0001
P=0.145
P=0.1419
P=0.1041
Resting Membrane Potential(mV)
20
0
-20
-40
-60
-80
-100
Control
MYOD
AB1167
ES
iPS
UiPSM_Day30
Myocytes

Figure S2 The expression of ectopic MYOD in UiPSMs

A. Representative images showed UiPSM and iMYOD differentiated myotubes. Scale bar,50uM.

B. Diameter of UiPSM and iMYOD differentiated myotubes. The myotube diameter of UiPSM derived myotubes was lower than MYOD mediated UiPSM derived myotubes. n = 3 independent experiments. (The 3 lines inside the violin plot represent medians and 95% confidence intervals.)

C. Box plot statistics showed the number of UiPSM and iMYOD differentiated myotubes. n = 3 independent experiments. (The centerline of the box plot represents the mean value)

D. Membrane excitability in UiPSM and iMYOD differentiated myocytes at day 30. Representative tracing of passive membrane depolarization evoked by a series of step currents (1 Hz by 3 ms) in UiPSM and iMYOD UiPSM-derived myogenic progenitors at day 30.

E. Violin plot showed the resting membrane potential of UiPSM and iMYOD differentiated myocytes at day 30. Then comparing with reported the means ± SE resting membrane potential (RMP) of myotubes within healthy (AB1167)[1] muscle tissue and human ES and iPS derived myocytes[2]. n = 3 independent experiments.

1 Nguyen, C. T., Ebrahimi, M., Gilbert, P. M. & Stewart, B. A. Electrophysiological analysis of healthy and dystrophic 3-D bioengineered skeletal muscle tissues. *American Journal of Physiology-Cell Physiology* **321**, C749-C759, doi:10.1152/ajpcell.00049.2021 (2021).

2 Skoglund, G. *et al.* Physiological and ultrastructural features of human induced pluripotent and embryonic stem cell-derived skeletal myocytes in vitro. *Proceedings of the National Academy of Sciences* **111**, 8275-8280, doi:10.1073/pnas.1322258111 (2014).

Figure S3

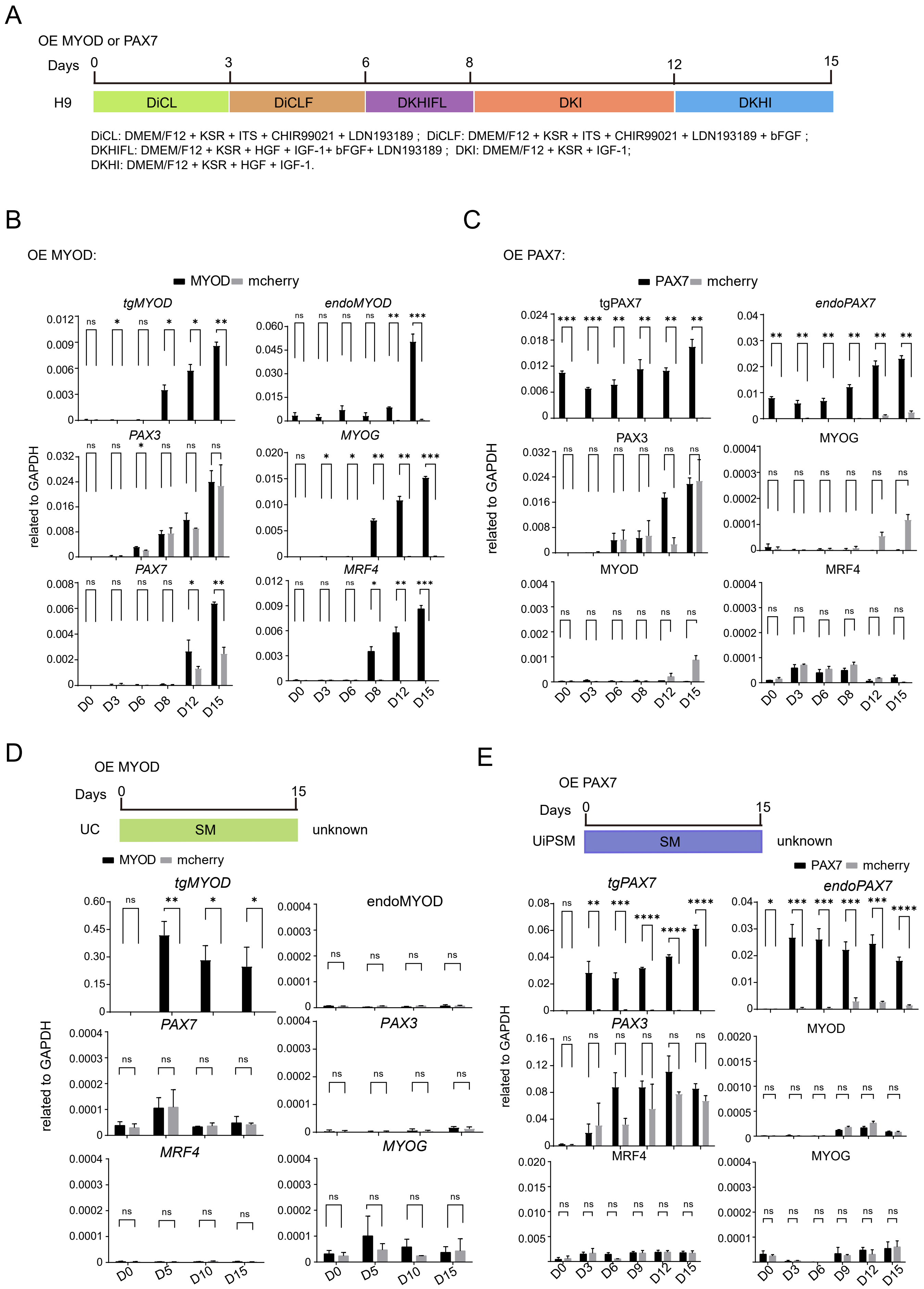

A
OE MYOD or PAX7
Days 0 3 6 8 12 15
H9
DiCL
DiCLF
DKHIFL
DKI
DKHI
DiCL: DMEM/F12 + KSR + ITS + CHIR99021 + LDN193189 ; DiCLF: DMEM/F12 + KSR + ITS + CHIR99021 + LDN193189 + bFGF ;
DKHIFL: DMEM/F12 + KSR + HGF + IGF-1+ bFGF+ LDN193189 ; DKI: DMEM/F12 + KSR + IGF-1;
DKHI: DMEM/F12 + KSR + HGF + IGF-1.
B
OE MYOD:
MYOD mcherry
tgMYOD
endoMYOD
PAX3
MYOG
PAX7
MRF4
related to GAPDH
D0 D3 D6 D8 D12 D15
C
OE PAX7:
PAX7 mcherry
tgPAX7
endoPAX7
PAX3
MYOG
MYOD
MRF4
related to GAPDH
D0 D3 D6 D8 D12 D15
D
OE MYOD
Days 0 15
UC
SM
unknown
MYOD mcherry
tgMYOD
endoMYOD
PAX7
PAX3
MRF4
MYOG
related to GAPDH
D0 D5 D10 D15
E
OE PAX7
Days 0 15
UiPSM
SM
unknown
PAX7 mcherry
tgPAX7
endoPAX7
PAX3
MYOD
MRF4
MYOG
related to GAPDH
D0 D3 D6 D9 D12 D15

Figure S3 The ectopic MYOD expressed in hESC(h9) and hUCs and the ectopic PAX7 expressed in hESC(h9) and UiPSMs.

A. Schematic overview of skeletal muscle myocytes differentiated from hESC(H9). This medium is also the base medium for optimizing myogenesis in UIPSM cells refering to Jérome Chal' paper [3]. The 15-day time window formulated the characteristic genes derived from the transcription factor MYOD-mediated myogenesis of UiPSM cells that had highly expressed in myoblasts.

B. The skeletal myocytes differentiated from hESC(H9) for 15 days when overexpressed ectopic *MYOD*. Representative gene expression of human skeletal muscle specific genes, including endo*MYOD, tgMYOD, PAX3, PAX7, MYOG, MRF4*. Mcherry as a negative control of overexpression vector. Data are mean ± SD, n = 3 independent experiments.

C. The skeletal myocytes differentiated from hESC(H9) for 15 days when overexpressed ectopic *PAX7*. Representative gene expression of human skeletal muscle specific genes, including endo*PAX7, tgPAX7, PAX3, MYOD, MYOG, MRF4*. Mcherry as a negative control of overexpression vector. Data are mean ± SD, n = 3 independent experiments.

D. The skeletal myocytes differentiated from human urine cells (UC) for 15 days when overexpressed *MYOD*. Representative gene expression of human skeletal muscle specific genes, including endo*MYOD, tgMYOD, PAX3, PAX7, MYOG, MRF4*. Mcherry as a negative control of overexpression vector. Data are mean ± SD, n = 3 independent experiments.

E. The skeletal myocytes differentiated from UiPSM cells for 15 days when overexpressed ectopic *PAX7*. Representative gene expression of human skeletal muscle specific genes, including endo endo*PAX7, tgPAX7, PAX3, MYOD, MYOG, MRF4*. Mcherry as a negative control of overexpression vector. Data are mean ± SD, n = 3 independent experiments.

3 Chal, J. *et al.* Generation of human muscle fibers and satellite-like cells from human pluripotent stem cells in vitro. *Nature Protocols* **11**, 1833-1850, doi:10.1038/nprot.2016.110 (2016).

Figure S4

A

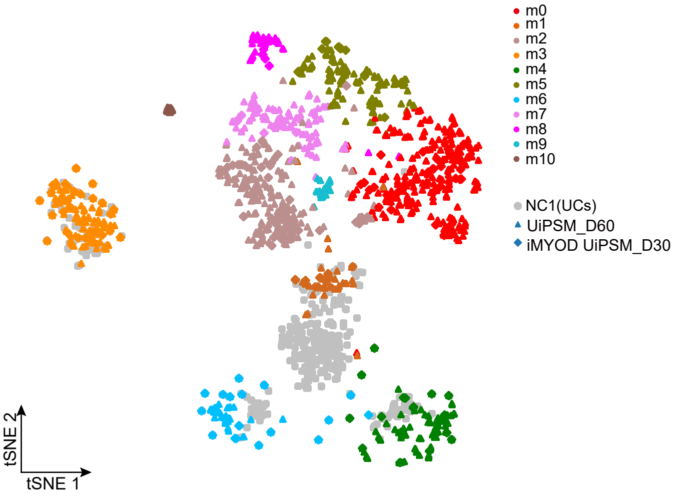

B

	Enriched GO Terms (David)	*P*-value
m0	striated muscle tissue development	6.72E-07
	muscle tissue development	1.41E-06
	skeletal muscle cell differentiation	4.32E-04
m3	muscle system process	2.46E-19
	muscle contraction	8.59E-18
	myofibril assembly	7.09E-16
	muscle cell development	1.14E-13
	skeletal muscle contraction	1.05E-10
m5	muscle tissue development	1.71E-12
	striated muscle tissue development	5.02E-11
	myofibril assembly	3.21E-07
	sarcomere organization	1.94E-05
	actin filament organization	4.19E-05
m6	striated muscle tissue development	8.50E-10
	striated muscle hypertrophy	3.87E-07
	actin filament bundle assembly	1.31E-05
	actin filament bundle organization	1.61E-05
m8	muscle organ development	3.41E-05
	striated muscle contraction	3.05E-04
	musculoskeletal movement	0.004295
	striated muscle cell apoptotic process	0.005437
m9	skeletal muscle tissue development	3.71E-10
	skeletal muscle cell differentiation	4.07E-08
	muscle filament sliding	0.008118421
	regulation of supramolecular fiber organization	0.010160038
	actin-myosin filament sliding	0.011689886

C

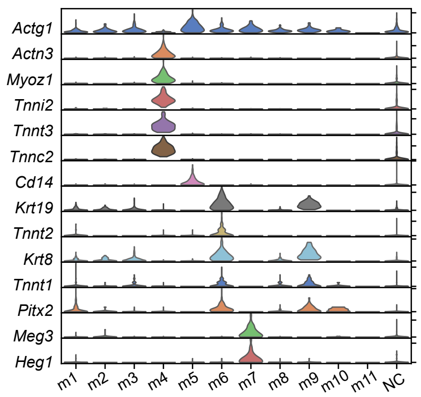

D

The number of cell mapping mm10

sample	NC1(UCs)	UiPSM_D60	iMYOD UiPSM_D30
cells	347	959	167

Figure S4 scRNA-seq analysis of the transplanted TA muscles when mapping mouse genome

A. t-SNE plot showed the projection of 1473 cells mapping to mouse genome(mm10). These cells could be divided in 11 color-coded clusters. The blocks, triangles and diamonds represent the control group (urine cells), and UiPSM and iMYOD UiPSM derived human myocytes, respectively.

B. Gene ontology (GO) analysis revealed gene expression profiles of the groups (m0, m3, m5, m6, m8, m9) that are highly associated with skeletal muscle development in the above 11 clusters. p value < 0.05.

C. Violin plot showing the expression and ratio of typical genes closed to skeletal muscle development in each cluster.

D. The number of cells of the grafts from transplanted UiPSM and iMYOD UiPSM derived human myocytes, mapped to mouse genome(mm10).

Figure S5

A

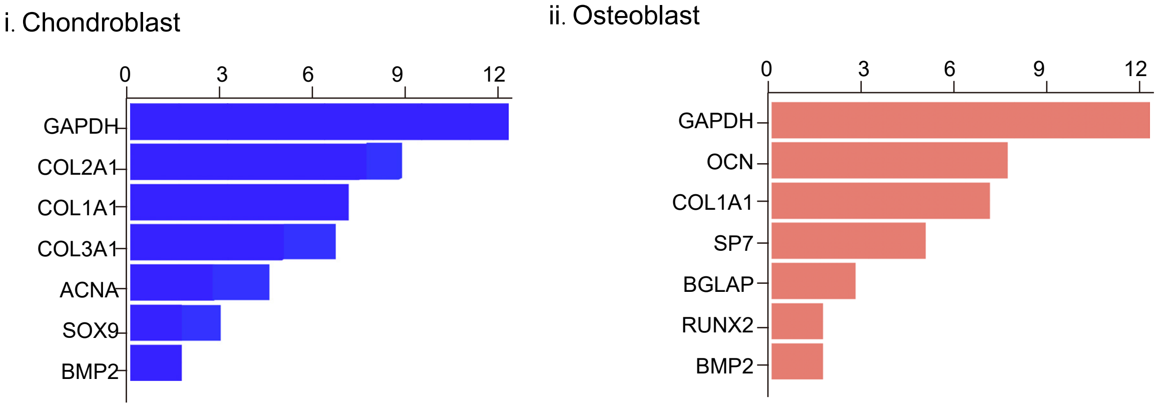

i. Chondroblast
0
3
6
9
12
GAPDH
COL2A1
COL1A1
COL3A1
ACNA
SOX9
BMP2
ii. Osteoblast
0
3
6
9
12
GAPDH
OCN
COL1A1
SP7
BGLAP
RUNX2
BMP2

B

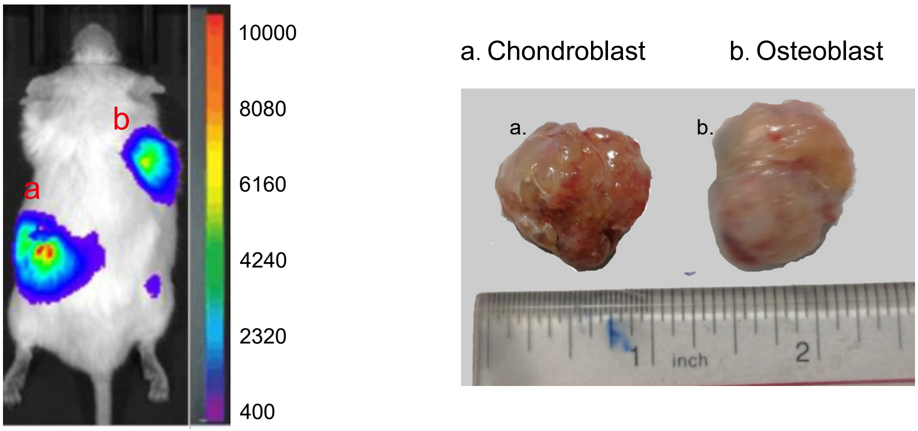

b
a
10000
8080
6160
4240
2320
400
a. Chondroblast
b. Osteoblast
a.
b.
1
inch
2

Figure S5 Transplantation of UiPSM cells-derived chondroblasts and osteoblasts in MITRG mice

A. The expression level of differential expression of representative genes in Chondroblast (i) and Osteoblast (ii), comparing with UiPSM cells. GAPDH as internal reference.

B. Luciferase labeled UiPSM cells-derived chondroblasts and osteoblasts were injected subcutaneously into the back of the MITRG mice. In vivo BLI signal in a representative MITRG mouse at days 30 after transplantation (left). Representative photograph of the anatomy of the above grafts (right). UiPSM derived chondroblast transplanted in to the left side of the mouse back (a) and UiPSM derived osteoblast transplanted in to the right side (b).

Table S1 Antibodies, Chemicals, and Recombinant Proteins; related to Experimental Procedures

REAGENT or RESOURCE	SOURCE	IDENTIFIER
**Antibodies**		
Mouse monoclonal anti- Human PAX3	R & D systems	Cat#MAB2457
Mouse monoclonal anti-Human PAX7	R & D systems	Cat#MAB1675
Mouse monoclonal anti-Human MYOD	R & D systems	Cat#MAB5966
Mouse monoclonal anti-Myosin Heavy Chain (MF20, MHC)	R & D systems	Cat#MAB4470
Mouse monoclonal anti-Human Laminin a3	R & D systems	Cat#MAB2144
Rabbit polyclonal anti-Human Desmin	Absin	Cat#abs106139
Rabbit polyclonal anti-Human MYOD	Cell signaling Technology	Cat#D8G3
Mouse monoclonal anti-Human HuNu(hNA)	Millipore	Cat#MAB1281
**Chemicals, and Recombinant Proteins**		
CHIR99021	Synthesized in GIBH	N/A
bFGF	PeproTech	GenPept: P09038
EGF	R & D systems	Cat#236-EG
EPZ5676	Selleck Chemicals	Cat#S7062
A8301	R & D systems	Cat#2939
SB431542	PeproTech	Cat#301836-41-9
Vitamin C	Sigma-Aldrich	Cat#49752
Dexamethasone Phosphate disodium (Dex)	Target Mol	Cat#T0947L
ITS	Gibco	Cat#41400045
IGF-1	Pepro Tech	Cat#250-19
HGF	R & D systems	Cat#294-HG-250
Bovine Serum Albumin (BSA)	Sigma	Cat# 9048-46-8
1-Thioglycerol	Sigma	Cat#96-27-5
β-Glycerophosphate	PeproTech	Cat#154804-51-0

Table S2 Primers for quantitative PCR; related to Experimental Procedures

GENE Name	Forward	Reverse
GAPDH	*GTGGACCTGACCTGCCGTCT*	*GGAGGAGTGGGTGTCGCTGT*
Total-MYOD	TGCCACAACGGACGACTT	GCAGGCCCACAGTAGGCA
Endo-MYOD	TAGCAGGTGTAACCGTAA	TGGCAAAGCAACTCTTAT
TgMYOD	GTACAGtGCAGGGGAAAG	TAGAAGTCGTCCGTTGTG
Total-PAX7	TGTGCCCTCAGGTTTAGT	GCTTCATCCTCCTCCTCTT
Endo-PAX7	TGGAGTGTTTGTTTGTTTGA	CCGTCTTCTTCGTCTTCT
TgPAX7	GTACAGtGCAGGGGAAAG	GCCATCTCCACTATCTTGT
PAX3	CTCCACGCTCCGGATAGTTC	ATCTTGTGGCGGATGTGGTT
MYF5	AATTTGGGGACGAGTTTGTG	CATGGTGGTGGACTTCCTCT
MYOG	GCTGTATGAGACATCCCCCTA	CGACTTCCTCTTACACACCTTAC
MYH3	TTGATGCCAAGACGTATTGCT	GGGGGTTCATGGCGTACAC
MRF4	CAACAATCCCTACGACTACGC	ACGTCAAAGGCACTATCGGTG
MYH7	GCAGTGTATGAGAGGATGT	CTCGTTGGTGAAGTTGATG
RUNX2	CTCACTACCACACCTACC	TTCATCCATTCTGCCACTA
BGLAP	AGCGAGGTAGTGAAGAGA	GATGTGGTCAGCCAACTC
BMP2	GTGGAATGACTGGATTGTG	TGATTAGTGGAGTTCAGATGA

SP7	CAGGCTATGCTAATGATTACC	GGCAGACAGTCAGAAGAG
OCN	TGGCCCTGAC TGCATTCTGC	GCTGTGCCGTCCATACTTTCG
OPN	CCTCTGAAGAAACGGATGACT	CTGTGTGTTTCCACGCTT
Collagen1	CGAGTATGGAAGCGAAGGTT	CACAAGCGTGCTGTAGGT
ACAN	AGGCAGCGTGATCCTTACC	GGCCTCTCCAGTCTCATTCTC
SOX9	GTACCCGCACTTGCACAAC	TCTCGCTCTCGTTCAGAAGTC
COL2A1	CGTCCAGATGACCTTCCTACG	TGAGCAGGGCCTTCTTGAG
COL9A1	TGTAAAACGACGGCCAGT	CAGGAAACAGCTATGACC
